# Characterization of bacteriophage vB_AbaS_SA1 and its synergistic effects with antibiotics against clinical multidrug-resistant *Acinetobacter baumannii* isolates

**DOI:** 10.1093/femspd/ftae028

**Published:** 2024-10-21

**Authors:** Sanaz Rastegar, Salehe Sabouri, Omid Tadjrobehkar, Ali Samareh, Hira Niaz, Nafise Sanjari, Hossein Hosseini-Nave, Mikael Skurnik

**Affiliations:** Medical Mycology and Bacteriology Research Center, Kerman University of Medical Sciences, Kerman, Iran; Department of Medical Microbiology (Bacteriology and Virology), Afzalipour School of Medicine, Kerman, Iran; Extremophile and Productive Microorganisms Research Center, Kerman University of Medical Sciences, Kerman, Iran; Department of Pharmaceutical Biotechnology, Faculty of Pharmacy, Kerman University of Medical Sciences, Kerman, Iran; Medical Mycology and Bacteriology Research Center, Kerman University of Medical Sciences, Kerman, Iran; Department of Medical Microbiology (Bacteriology and Virology), Afzalipour School of Medicine, Kerman, Iran; Department of Biochemistry, School of Medicine, Kerman University of Medical Sciences, Kerman, Iran; Department of Bacteriology and Immunology, Human Microbiome Research Program, Faculty of Medicine, University of Helsinki, Helsinki, Finland; Medical Mycology and Bacteriology Research Center, Kerman University of Medical Sciences, Kerman, Iran; Medical Mycology and Bacteriology Research Center, Kerman University of Medical Sciences, Kerman, Iran; Department of Medical Microbiology (Bacteriology and Virology), Afzalipour School of Medicine, Kerman, Iran; Department of Bacteriology and Immunology, Human Microbiome Research Program, Faculty of Medicine, University of Helsinki, Helsinki, Finland

**Keywords:** *Acinetobacter baumannii*, bacteriophage, drug resistance, time-kill, synergistic effect, siphovirus

## Abstract

*Acinetobacter baumannii* is a major cause of nosocomial infections globally. The increasing prevalence of multidrug-resistant (MDR) *A. baumannii* has become an important public health concern. To combat drug resistance, alternative methods such as phage therapy have been suggested. In total, 30 MDR *A. baumannii* strains were isolated from clinical specimens, and their antibiotic susceptibilities were determined. The *Acinetobacter* phage vB_AbaS_SA1, isolated from hospital sewage, was characterized. In addition to its plaque size, particle morphology, and host range, its genome sequence was determined and annotated. Finally, the antibacterial effects of phage alone, antibiotics alone, and phage/antibiotic combinations were assessed against the *A. baumannii* strains. Phage vB_AbaS_SA1 had siphovirus morphology, showed a latent period of 20 min, and a 250 PFU/cell (plaque forming unit/cell) burst size. When combined with antibiotics, vB_AbaS_SA1 (SA1) showed a significant phage-antibiotic synergy effect and reduced the overall effective concentration of antibiotics in time-kill assessments. The genome of SA1 is a linear double-stranded DNA of 50 108 bp in size with a guanine-cytosine (GC) content of 39.15%. Despite the potent antibacterial effect of SA1, it is necessary to perform additional research to completely elucidate the mechanisms of action and potential constraints associated with utilizing this bacteriophage.

## Teaser

A novel bacteriophage was isolated from hospital wastewater; its genome was analyzed, and when tested with antibiotics, it showed effective results against an MDR *A. baumannii*. This study could help with treatments for infections.

## Introduction

*Acinetobacter baumannii* causes different hospital-acquired infections, including meningitis, endocarditis, ventilator-associated pneumonia, bacteremia, urinary tract infections, gastrointestinal infections, skin infections, and surgical wound infections (Michalopoulos and Falagas 2010). This bacterium is one of the most problematic pathogens among the nosocomial infection-causing ESKAPE group of pathogens (*Enterococcus faecium, Staphylococcus aureus, Klebsiella pneumoniae, A. baumannii, Pseudomonas aeruginosa*, and *Enterobacter* spp.) due to its resistance even to the last-line antibiotics such as colistin, tigecycline, and carbapenems (Galac et al. 2020).

*Acinetobacter baumannii* has been recognized as a global threat because of its ability to acquire multidrug-resistance (MDR), extended drug resistance (XDR), and pan-drug resistance (PDR) (Harding et al. 2018). ‘MDR *Acinetobacter* spp.’ are defined as strains that resist at least one agent in three or more antimicrobial categories. These categories include aminoglycosides, antipseudomonal carbapenems (such as imipenem, meropenem, and doripenem), antipseudomonal fluoroquinolones (like ciprofloxacin and levofloxacin), antipseudomonal penicillins combined with β-lactamase inhibitors (e.g. piperacillin-tazobactam and ticarcillin-clavulanic acid), extended-spectrum cephalosporins, folate synthesis pathway inhibitors, penicillins combined with β-lactamase inhibitors, polymyxins, and tetracyclines. XDR isolates are those that are non-susceptible to at least one agent in all but two or fewer antimicrobial categories previously listed. PDR refers to organisms that are non-susceptible to all antimicrobial agents listed (Magiorakos et al. 2012).

While there is no consensus recommendation regarding the optimal treatment of MDR and XDR *A. baumannii* infections, several antibiotics, including colistin, ampicillin-sulbactam, and meropenem, have been used as monotherapy or combination therapy. Unfortunately, isolates that are resistant to commonly prescribed antibiotics lead to a poor prognosis with a high rate of treatment failure and significantly increased mortality (Kengkla et al. 2018, Liu et al. 2021, Mohebi et al. 2023). As such, *A. baumannii* is classified as a bacterium requiring urgent research for effective treatments. Consequently, various investigations have been conducted to address microorganism-caused diseases through innovative approaches (Samare-Najaf et al. 2023, Hajinezhad et al. 2024). The use of phages, or combination treatment with phages and antibiotics, has recently been considered a promising approach to deal with these issues (Kengkla et al. 2018).

Bacteriophages, or phages, are viruses that infect bacteria. Phages are considered the most abundant biological entities on Earth, with over 10^30^ estimated individual virions on our planet (Lara ). Although phages can be isolated from almost any source, sewage is one of the richest sources, particularly in hospital environments, where wastewater and sewage directly related to hospitals serve as primary sources (Weber-Dąbrowska et al. 2016). Phages can follow either a lytic (virulent) or a lysogenic (temperate) life cycle; a typical phage lytic cycle of infection is characterized by adhesion to the bacterial cell via recognition of host surface receptors, injection of the phage genome into the bacterial cytosol, viral replication, and protein synthesis, followed by host lysis and release of new phage particles (Weber-Dąbrowska et al. 2016, Kakasis and Panitsa 2019, Grygorcewicz et al. 2020). A lysogenic phage infects a host cell and either integrates its nucleic acid into the host genome or exists as a plasmid within the host cell, where it remains stable as a prophage for many generations. The prophage can be induced to leave the cell as a lytic phage under certain conditions, such as the presence of antibiotics (Blasco et al. 2019). Most bacteriophages are highly specific for one or a few strains/species of related bacteria. The significant differences between bacterial prokaryotic and human eukaryotic cells prevent cross-infection (Koskella and Brockhurst 2014). The lytic cycle of infection begins with the adsorption of the phage to the host cell by binding to specific receptors, including capsules, teichoic acids, membrane proteins, and lipopolysaccharides (Bertozzi Silva et al. 2016). Phages appear safe for humans, and their use in healthy volunteers has no side effects (Grygorcewicz et al. 2020). After the discovery of bacteriophages, the idea of phage therapy was developed, but it was almost forgotten after the discovery of antibiotics. However, in recent years, phage therapy has received new interest owing to increased antibiotic resistance and technical advances in purifying bacteriophages (Kakasis and Panitsa 2019).

Phage therapy has distinct advantages over traditional antibiotic treatment because phages are (i) naturally occurring antibacterial agents, (ii) self-replicating, (iii) self-limiting after resolution of infection, (iv) active against both MDR and antibiotic-sensitive bacteria, (v) particular with low inherent toxicity, (vi) able to co-evolve with bacteria, and (vii) able to penetrate biofilms (Chang et al. 2018). The emergence of bacterial resistance to phages is possible because bacteria have or can develop multiple mechanisms to prevent viral infection. The emergence of bacterial resistance to phages can be mitigated by using phage cocktails, administering higher initial doses of phages, or combining them with antibiotics (Principi et al. 2019).

Phages remain effective against MDR pathogens because they use a completely different mechanism to kill bacteria than antibiotics. Therefore, they can also be used with antibiotics (Lin et al. 2017). Such phages and antibiotic therapy combinations can exhibit synergistic antibacterial effects, a phenomenon known as phage-antibiotic synergy (PAS) (Liu et al. 2022). PAS has been observed to reduce the emergence of both phage-resistant and antibiotic-resistant strains successfully (Zhang et al. 2022). In various studies, PAS suggests that such combination therapy could be effective against multidrug-resistant bacteria such as *A. baumannii*. Furthermore, the integration of phages with other antimicrobial substances, employing delivery and encapsulation techniques for phages, and utilizing genetically modified phages along with endolysins extracted from phages have all exhibited promising results (Tan et al. 2022, Li et al. 2023).

Although numerous *A. baumannii* phages have been isolated in recent years, the majority of these phages lack adequate characterization. Given the phages’ high specificity and their susceptibility to resistance development, there is an ongoing need to isolate and identify new phages. This study aimed to isolate, characterize, and *in vitro* apply a new phage specific to MDR *A. baumannii* strains.

## Materials and methods

### Bacterial isolates

This study obtained non-duplicate clinical isolates of *A. baumannii* from hospitalized patients in Afzali-Pour Hospital, Kerman, Iran, from February to September 2021. The bacterial isolates were incubated on eosin methylene blue agar plates (Merck, Germany) overnight at 37°C. Biochemical tests were done to identify the isolates, including catalase, oxidase, sulfide indole motility (SIM) and triple sugar iron (TSI), and lysine decarboxylase (Ghahraman et al. 2020). Finally, the biochemically identified isolates were verified through polymerase chain reaction amplification of the 16s rDNA and *blaOXA-51* gene (Hashemizadeh et al. 2022).

### Antimicrobial sensitivity test

Antimicrobial susceptibility was determined by the disk diffusion method according to the Clinical and Laboratory Standards Institute (CLSI) guideline (CLSI 2021). Imipenem (10 µg), meropenem (10 µg), amikacin (30 µg), ciprofloxacin (5 µg), cefepime (30 µg), trimethoprim/sulfamethoxazole (1.25/23.75 µg), cefotaxime (30 µg), ceftriaxone (30 µg), levofloxacin (5 µg), ampicillin/sulbactam (20 µg), gentamicin (10 µg), and ceftazidime (30 µg) were used. *Pseudomonas aeruginosa* ATCC 27853 and *Escherichia coli* ATCC 25922 were used for quality control in this test. The isolates resistant to at least three antimicrobial agent classes were considered MDR (Magiorakos et al. 2012).

### Determination of MIC

The minimal inhibitory concentration (MIC) of each antibiotic (colistin, ampicillin-sulbactam, and meropenem) was determined in three independent experiments using a standard microdilution procedure in 96-well microtiter plates. Briefly, different concentrations of the indicated antibiotics were prepared by performing 2-fold serial dilutions and added to an equal volume (100 µl) of bacterial culture in Mueller Hinton broth in each well of the 96-well microplates. Microplates were incubated at 37°C for 18 h. The following steps were taken to determine the MIC value for each antibiotic: 20 µl of 0.1% solution of 2,3,5-triphenyl tetrazolium chloride (TTC) was added to each well, followed by further incubation of the microplates for 2–3 h at 37°C to allow viable bacterial cells to convert TTC to red formazan through dehydrogenase. MIC was determined as the minimum antibiotic concentration required to inhibit formazan production. The determination of MIC values was carried out for each antibiotic individually and in combination with phage (Kim et al. 2018, Nikolic et al. 2022). *Pseudomonas aeruginosa* ATCC 27853 and *E. coli* ATCC 29522 were used for quality control in this test.

### Phage isolation and enrichment

Hospital wastewater samples were collected from Afzali-Pour Hospital in Kerman, Iran, from January to September 2021. Samples were collected in sterile tubes and centrifuged at 5000 × *g* for 10 min to remove large particles. The supernatant was filtered using 0.22 µm syringe filters (Startech, Taiwan) and stored at 4°C. Five random MDR *A. baumannii* isolates (Ab3, Ab4, Ab5, Ab8, and Ab9) were grown in Luria-Bertani (LB) broth medium (HiMedia, India) as the host for phage isolation. Then, 10 ml of each sample supernatant was added to 100 ml exponential growth cultures (in the early logarithmic phase determined by OD_600_ of 0.4–0.6) supplemented with 0.1 M calcium chloride (Merck, Germany). The cultures were incubated in a shaking incubator at 37°C and 40 rpm for 48 h. Next, the samples were centrifuged at 12 000 × *g* for 15 min, and the supernatant was filtered through a 0.22 µm pore-sized membrane syringe filter to separate phages from other contaminants. The filtrate was used for the double-agar overlay method. Briefly, 200 µl of the early log phase bacterial culture (OD_600_ ≈ 0.4) and 200 µl of filtrate supernatant were mixed and incubated for 15 min at 37°C for proper absorption. The medium was mixed with 3 ml of LB soft agar containing 0.7% agar and was poured onto the lower agar, followed by swirling to create a uniform upper layer. The plates were incubated at 37°C overnight. Zones of lysis or plaque formation after incubation indicated the presence of lytic phages. The clear phage plaques were picked up from the plate for further purification, and the phage titer was determined (Manohar et al. 2019).

### Plaque purification of phages

Each plaque formed on the plate surface contains ∼10^5^–10^6^ phages. The established plaque was dissolved in 1.5 ml of LB medium. Then, 200 µl of chloroform was added to eliminate bacterial contamination according to the procedure mentioned in the phage isolation section. The plaque was purified three times for each phage to obtain homogenous phage lysates. The titers of the phages isolated against MDR *A. baumannii* strains were expressed as plaque-forming units (PFU) per ml (PFU/ml), as described by Carlson and Miller (Habibinava et al. 2022).

### Phage morphology by TEM

For a detailed study of the structure and morphology of the phage, 10 µl of purified phage suspension (10^8^ PFU/ml) was deposited on a carbon-coated copper grid for 30 s and stained negatively with 2% uranyl acetate (w/v) for 1 min. After drying, phage particles were observed using transmission electron microscopy (TEM; ZEISS EM 900, Germany) at an acceleration voltage of 50 Kv (Bagińska et al. 2023). The phage family was detected by morphological characteristics using the latest changes in the International Virus Classification Committee (ICTV) report (https://ictv.global/taxonomy).

### Host range investigation

Spot tests determined the host ranges of the phages against 30 clinical isolates of *A. baumannii*, five clinical isolates of *K. pneumoniae*, five clinical isolates of *E. coli*, and five clinical isolates of *P. aeruginosa*. The *K. pneumoniae, E. coli*, and *P. aeruginosa* strains originated from the bacterial strain collection at the Microbiology and Virology Department of Kerman University of Medical Sciences. A 200 µl aliquot of an overnight bacterial broth culture (10^9^ CFU/ml) was mixed with melted soft agar medium and spread on a plate containing solidified agar medium to make a double-layer agar. A 5 µl drop of phage stocks was spotted on each plate. The plates were incubated at 37°C overnight. The zones of clearing were checked for the antibacterial activity of the phages (Topka-Bielecka et al. 2020).

### The efficiency of plating

To determine the efficiency of the phage in infecting the bacterial strains, 5 µl drops of serial dilutions of the phages (10^10^–10² PFU) were spotted on seeded double-layer agar plates (prepared as described above). After overnight incubation of the plates at 37°C, lysis or individual plaques within the spots were checked (Regeimbal et al. 2016). The efficiency of platings (EOPs) (mean PFU on target bacteria/mean PFU on host bacteria) and the standard deviation for the three measurements were calculated. With EOP values ≥ 0.5, the phage infection efficiency was considered high for a particular phage–bacterium combination. EOP values between 0.1 and 0.5 indicated medium efficiency, and between 0.001 and 0.1, low efficiency. An EOP ≤ 0.001 was classified as inefficient (Khan Mirzaei and Nilsson 2015).

### Determination of the optimal multiplicity of infection

To determine the optimal multiplicity of infection (MOI), 100 µl of the host strain (10^4^ CFU/ml) was added to 96-well microtiter plates and mixed with 100 µl of different dilutions of phage suspensions (10^7^, 10^6^, 10^5^, 10^4^, 10^3^, and 10^2^ PFU/ml; MOIs 1000, 100, 10, 1, 0.1, and 0.01, respectively). The OD_600_ of each well was measured with a microplate ELISA reader (ELX800, BioTek Instruments, USA) at 0, 2, 4, 6, 8, and 24 h post-infection. The wells containing only the host strain or phage particles were used as controls. The experiment was performed in triplicate (Birge 2013, Torabi et al. 2021).

### One-step growth curve

A one-step growth experiment was carried out to determine the latent period (the interval between the adsorption of the phage to the bacterial cell and the release of the phage progeny) and the burst size (the ratio of the final count of released phage particles to the count of the infected bacterial cells during the latent period) of the phage. Host bacteria were grown in 10 ml LB broth to the early log phase. Then, the cultured bacteria were centrifuged at 6000 × *g* for 5 min, the supernatant was discarded, and the pellet was resuspended in 1 ml LB broth. The phage suspension was mixed with bacterial suspension according to the MOI 0.1, and the culture was incubated for 15 min at room temperature (RT) to allow phage adsorption. After the incubation, the non-adsorbed phages were removed by centrifugation at 10 000 *g* for 1 min, and the pellet containing the adsorbed phages was suspended in 10 ml of LB broth. The phage titer in the culture was determined every 10 min (Ghaznavi‐Rad et al. 2022).

### Phage propagation using the Gabrichevsky method

To obtain a high titer lysate of phage vB_AbaS_SA1, the Gabrichevsky method was used (Gomez-Raya-Vilanova et al. 2022). Briefly, in this method, a 175-cm^2^ cell culture bottle was prepared by casting into it as a wedge 100 ml 2% LB agar. *Acinetobacter baumannii* strain Ab6 was cultured in 5 ml LB medium at 37°C with shaking at 150 rpm. After 1 h of incubation, the OD_600_ of the culture was adjusted to 0.1, and 2.33 ml was flooded evenly on the agar in the cell culture bottle. The bottle was incubated for 3 h at 37°C, the excess liquid was removed, and 2.33 ml of phage vB_AbaS_SA1 (10^7^ PFU/ml) was flooded evenly on the agar. The bottle was incubated overnight (16 h) at 37°C. The phages and bacteria on the agar surface were then collected with 10 ml of SM buffer by shaking at RT for 20 min. The bottle was allowed to stand upright for 1 h, and the collected liquid was transferred to a 50 ml Falcon tube to which 200 µl of chloroform was added. After incubation for 30 min, it was centrifuged at 5000 rpm for 10 min, and the supernatant was filtered through a 0.2 µm syringe filter. The phage titer was measured using the double-layer agar assay.

### DNA isolation

The phage vB_Ab_SA1 DNA was isolated from the high-titer phage lysate using the phenol-chloroform method (Sambrook and Russell 2006). Briefly, 1.3 µl DNase I (1 U/ul) and 4 µl RNase A (1 mg/ml) were added to 400 µl of vB_Ab_SA1 phage lysate and incubated for 1 h at 37°C. Then 16 µl of 0.5 M EDTA, 1.2 µl of Protease K (20 mg/ml), and 20 µl of 10% SDS were added, and the tube was incubated at 65°C for 60 min. After cooling to RT, 1 volume of phenol was added and mixed gently by vortexing for 10–15 min, followed by brief centrifugation to separate the phases. The phenol extraction of the water phase was repeated two to three times, followed by extraction with 1 volume of chloroform. After adding 0.1 volume of 3 M sodium acetate (pH 7.0), the DNA was precipitated with 2 volumes of absolute EtOH and pelleted by centrifugation for 5 min at 13 rpm. The supernatant was discarded, and the pellet was washed with 1 ml of 70% ethanol. After centrifugation at RT for 15 min at 13 rpm, the supernatant was carefully decanted, and the pellet was air-dried at 37°C. The pellet was dissolved into MiQ water by incubating overnight at 4°C. The DNA concentration was determined using the Qubit™ 4 Fluorometer device (Invitrogen, Thermo Fisher Scientific).

### Genome sequencing and bioinformatic analysis

The genomic DNA was sequenced at Novogene (https://www.novogene.com) using the Illumina PE150 platform. The quality of the obtained 9.3 million sequence reads was checked using FastQC v.0.12.1 (http://www.bioinformatics.babraham.ac.uk/projects/fastqc/). The de novo assembly, using a 50 000 read subset of forward and reverse sequence reads was carried out at the Chipster platform using the A5-miseq pipeline (Kallio et al. 2011, Coil et al. 2014). To confirm the assembly, all the original reads were mapped to the assembly contigs using the Geneious Prime 2022.2.2 (https://www.geneious.com/). All the sequence reads were used to identify the physical ends of the genome using the PhageTerm software (Garneau et al. 2017). Preliminary annotation was performed using RAST (Rapid Annotation using Subsystem Technology) (Aziz et al. 2008). The annotation was manually edited and checked using Artemis 18.2.0 (Carver et al. 2012), the MPI bioinformatics toolkit Hhpred (Alva et al. 2016), BLASTp (Protein Basic Local Alignment Search Tool) (Altschul et al. 1990), and Pharokka (Bouras et al. 2023). The phage life cycle was determined using webtool Phage AI (https://phage.ai/) (Tynecki et al. 2020). Antibiotic resistance genes were found using ResFinder4.1 (Bortolaia et al. 2020), and virulence genes were determined using VirulenceFinder 2.0 (https://bio.tools/virulencefinder) (Malberg Tetzschner et al. 2020). tRNA was predicted using tRNAscan-SE (http://lowelab.ucsc.edu/tRNAscan-SE/) (Chan et al. 2021). The whole genome tree was constructed using ViPTree (https://www.genome.jp/viptree/) (Nishimura et al. 2017), and the phylogeny compared with vieuviruses was performed by VICTOR (Meier-Kolthoff and Göker 2017).

### Restriction endonuclease analysis

To confirm the genome assembly, phage DNA was digested using restriction enzymes EcoRI and Kpnl (Thermo Fischer Scientific) that in *in silico* digestion using the NEBcutter (https://nc3.neb.com/NEBcutter/) produced well-separated bands. The restriction digestions were carried out in a total volume of 20 µl containing 1 µl of phage DNA, 1 µl of restriction enzyme, and 2 µl of 10 × Fast digest buffer for 2 h at 37°C. The digested DNA fragments were separated in 0.7% agarose gel containing Midori green and imaged using the Bio-Rad GelDoc XR + imaging system.

### Time-kill curve analysis for assessing the synergism between the phage SA1 and antibiotics

The bacteriophage–antibiotic interactions were examined using the MIC method in the 96-well plate, modified to this specific combination. MIC values for three antibiotics (colistin, ampicillin-sulbactam, and meropenem) were measured as explained above. The experiment was repeated using a combination of antibiotics and phage SA1 (only for 10 *A. baumannii* isolates that were sensitive to phage SA1). Throughout the testing process, the concentrations of the antibiotics varied, whereas the concentration of phage SA1 remained constant at a sub-inhibitory level (10^5^ PFU/ml, MOI 0.1). Then, the reduction in MIC values was evaluated. The effectiveness of the combination of phage SA1 and each antibiotic was confirmed using the time-kill curve method on three isolates (Ab1: ampicillin/sulbactam, Ab2: colistin, and Ab6: meropenem). Changes in the bacterial counts were monitored in parallel in test tubes with the following contents: bacteria only (∼10^6^ CFU/ml) as control; bacteria and antibiotics (sub-MIC); bacteria and phages SA1 (10^5^ PFU/ml = MOI 0.1); and bacteria, phages SA1 (10^5^ PFU/ml), and antibiotics (sub-MIC). The test tubes were incubated at 37°C for 24 h, and bacterial counts were determined after 0, 4, 8, and 24 h of incubation by the agar dilution plate counting method. These plates were incubated overnight at 37°C, and bacterial colonies were counted. The experiment was performed in triplicate, and the average was expressed as logarithms with corresponding standard errors (mean SE). Synergy was defined as a 2-log_10_ CFU/ml kill compared to the most effective agent (or double-combination regimen) alone at 24 h. Bactericidal activity was described as a 3-log_10_ CFU/ml reduction from baseline (Knezevic et al. 2013, Coyne et al. 2022, Nikolic et al. 2022).

### Statistical analysis

The results were presented as Mean ± SEM. GraphPad Prism 8.0 and SPSS version 20 software were used for data analysis. Two-way ANOVA was used to compare the groups, and a *P*-value < .05 was considered statistically significant.

## Results

### Antibiotic susceptibility and MICs of clinical *A. baumannii* isolates

For 30 MDR *A. baumannii* isolates, disk diffusion and broth microdilution tests (for colistin) were performed. The results showed that all the isolates were resistant to the meropenem, imipenem, amikacin, gentamicin, ciprofloxacin, levofloxacin, ceftazidime, cefepime, and ceftriaxone. The majority of the isolates were resistant to cefotaxime (96.7%), trimethoprim/sulfamethoxazole (90%), and ampicillin-sulbactam (73.3%). Only one *A. baumannii* isolate (3.3%) was resistant to colistin. Additionally, for the 10 MDR *A. baumannii* isolates (that were cleared to be susceptible to isolated phage in coming steps), MIC tests were also conducted for two antibiotics: ampicillin-sulbactam and meropenem. These findings are presented in Table 1.

**Table 1.** Multidrug resistant isolates of *A. baumannii* were used in this study, and their MICs for antibiotics ampicillin/sulbactam, meropenem, and colistin were tested in the absence or the presence of the phage.

Isolate number	MIC of meropenem (µg/ml)	MIC of meropenem (µg/ml) + phageSA1	MIC of colistin (µg/ml)	MIC of colistin (µg/ml) + phageSA1	MIC of ampicillin-sulbactam (µg/ml)	MIC of ampicillin-sulbactam (µg/ml) + phage SA1
Ab1	128	16	0.5	0.06	128	16
Ab2	16	4	1	0.25	64	16
Ab3	64	32	0.5	0.25	32	16
Ab4	32	8	1	0.05	64	32
Ab5	64	32	0.25	0.06	256	32
Ab6	128	4	0.5	0.25	64	32
Ab7	32	2	0.5	0.06	128	32
Ab8	64	32	0.12	0.06	128	32
Ab9	16	4	4	2	8	4
Ab10	16	4	0.12	0.06	128	16

### Phage isolation

**Figure 1.**
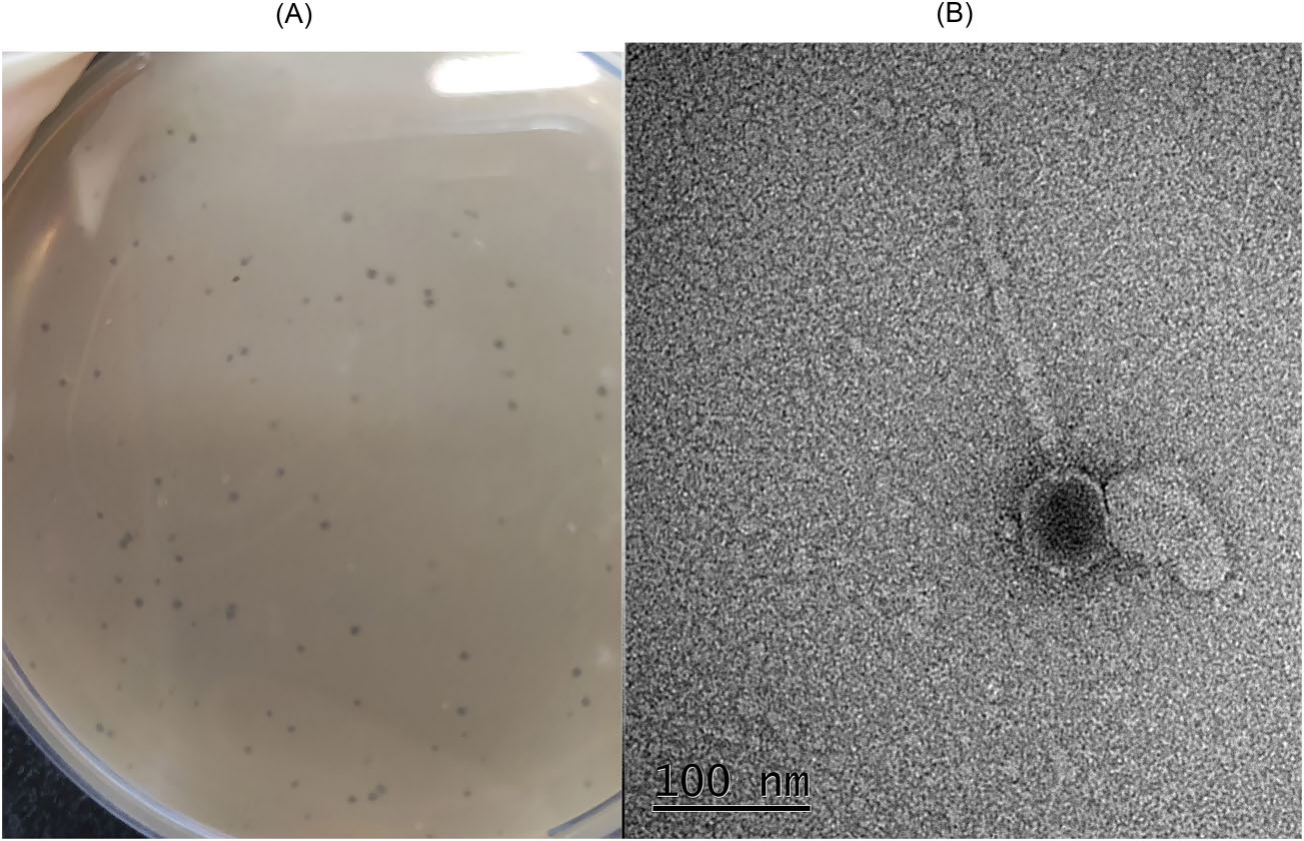
(A) Clear plaque formed by phage vB_AbaS_SA1 on *A. baumannii* host lawn on a double-layer agar plate. (B) Virion particle morphology of phage vB_AbaS_SA1 observed by TEM.

Phage SA1 (named vB_AbaS_SA1 after characterization) was isolated from the sewage of Afzali-Pour Hospital in Kerman. The phage formed clear plaques of ∼0.76–1 mm in diameter on bacterial lawns (Fig. 1A).

### Phage morphology

The morphology of phage particles was determined by the TEM. The head diameter and length are 56.3 ± 1.2 nm and 78.4 ± 3.9 nm, respectively, and the non-contractile tail length is 246.6 ± 6.1 nm (Fig. 1B). Thus, the phage is morphologically a siphovirus with characteristic icosahedral head, long non-contractile tail, and double-stranded DNA. The phage was named based on phage morphology and its host *A. baumanii* as vB_AbaS_SA1 (phage SA1).

### Phage host range

The host range of vB_AbaS_SA1 was tested against clinical isolates of MDR *A. baumannii* (N: 30), *K. pneumoniae* (N: 5), *E. coli* (N: 5), and *P. aeruginosa* (N: 5). The infectivity was categorized based on plaque formation. Ten out of 30 (33%) MDR *A. baumannii* isolates were sensitive to vB_AbaS_SA1, as shown in Table S1. None of the clinical isolates of *K. pneumoniae, E. coli*, and *P. aeruginosa* were susceptible to the isolated phage.

### Efficiency of plating

*Acinetobacter baumannii* isolates with a positive result in the spot test were subjected to the EOP test. Phage vB_AbaS_SA1 showed a highly productive infection with an EOP value ≥ 0.5 for five out of 10 strains. One isolate had medium efficiency (EOP between 0.1 and 0.5), and four isolates had low efficiency (EOP values between 0.001 and 0.1) As shown in Table S2.

### Optimal multiplicity of infection

We tested the MOI, ranging from 0.001 to 1000, to determine the optimal amounts of phage particles to eradicate or decrease the host cell when the bacterial starting concentration was 10^4^ CFU/ml. When the ratio of vB_AbaS_SA1 to the host (*A. baumannii* Ab8) was 10, the medium turbidity was the lowest, and this ratio was considered the optimal MOI. This optimal MOI was then used in subsequent experiments, as demonstrated in Fig. S1.

### One-step growth curve

**Figure 2.**
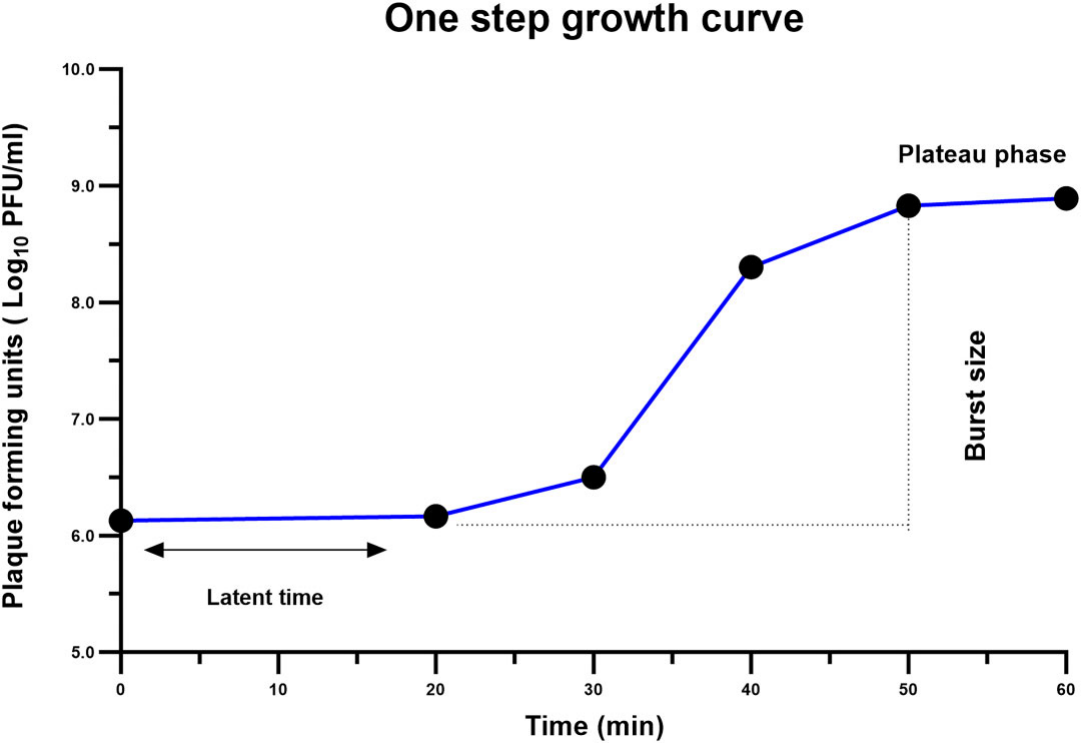
One-step growth curve of the phage vB_AbaS_SA1. Data are shown as mean ± SD.

Based on the one-step growth curve of the phage, the latent period was 20–22 min, and the plateau phase was reached after 50 min. The phage’s burst size was 250 PFU per infected cell (Fig. 2).

### Analysis of the vb_AbaS_SA1 phage genome

The vb_AbaS_SA1 genome is linear double-stranded DNA of 50 108 bp in size with a GC content of 39.15%. Approximately 77% of the 9.3 million sequence reads mapped back to the 50 kb contig. The PhageTerm analysis did not identify any distinct phage termini (Fig. S2), so the genome’s left end was set upstream of the gene encoding the terminase small subunit. The genome features of vB_ AbaS _SA1 are shown in Table 2.

**Table 2.** vB_AbaS_SA1 genome characterization.

Class	Caudoviricetes
Genus	Vieuvirus
Genome length(bp)	50 108 bp
GC content (%)	39.15
Predicted genes	73
tRNA	None
Repeat region	None
Genes for temperate lifecycle	1
Antibiotics resistance genes	None
Virulence genes	None

**Figure 3.**
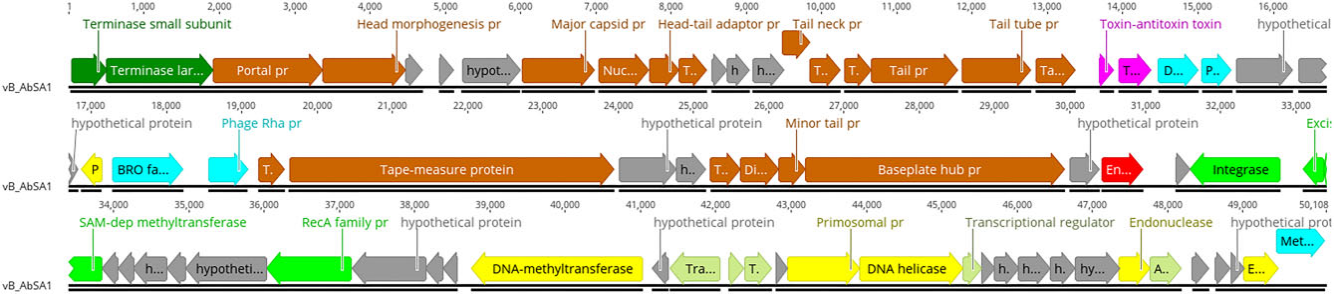
The genome organization of phage vB_AbaS_SA1 (GenBank accession number: BankIt2791774 vB_AbaS_SA1 PP236949). The different functional groups are indicated with colors: dark green, terminases; brown, phage structural proteins; purple, toxin-antitoxin system; gray, hypothetical proteins; yellow, nucleic acid metabolism; light green, lysogeny control; red, endolysin; turquoise, miscellaneous. The figure was generated using Geneious Prime vs 2023.2.1.

Annotation results showed that the vB_AbaS_SA1 genome contains 73 predicted genes, 58 in forward and 15 in reverse orientation (Fig. 3). Of 73 genes, 30 (41%) were annotated to encode hypothetical proteins and 44 (59%) to encode proteins with predicted functions.

**Figure 4.**
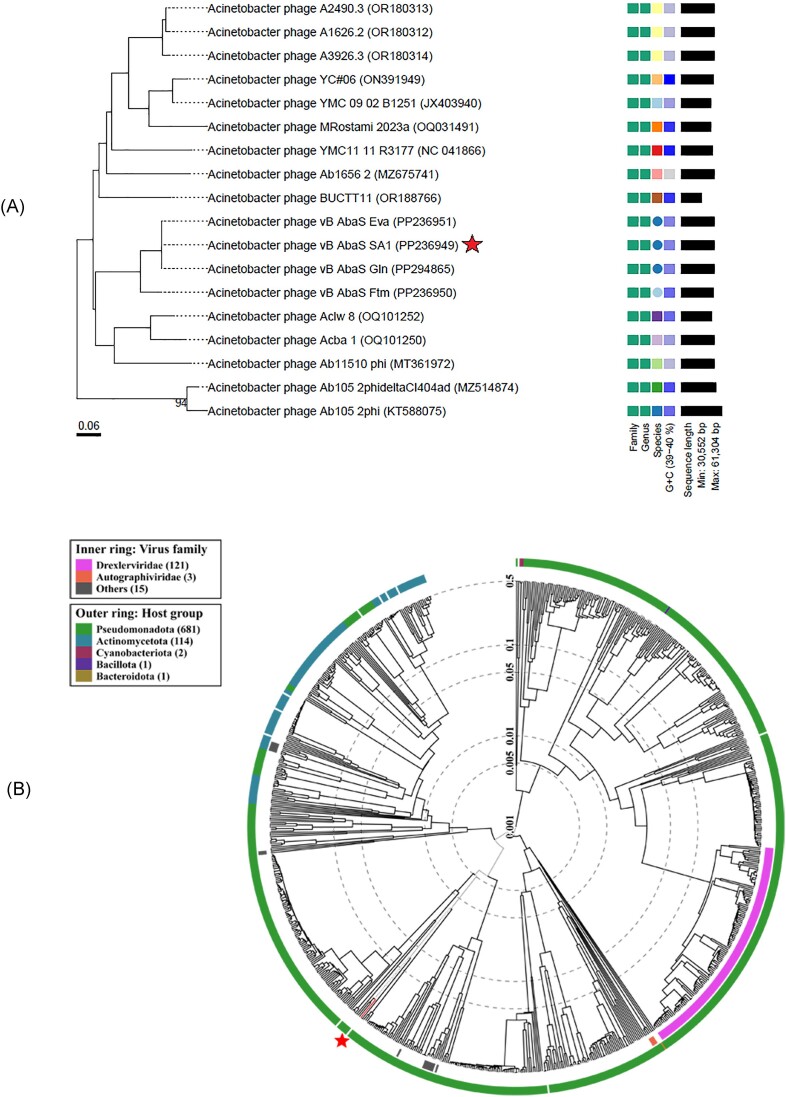
(A) Segment of the rectangular proteomic tree showing the bacteriophages closest in relation to vB_AbaS_SA1, (B) circular proteomic tree depicting prokaryotic dsDNA viruses with colors indicating virus families and host groups.

The phylogenetic proteomic tree generated by VICTOR (Fig. 4) showed that vB_AbaS_SA1 is closely related to two *Acinetobacter* phages (accession numbers PP236951 and PP294865). Based on the genome analysis, phage vB_AbaS_SA1 is one species of genus *Vieuvirus* in class *Caudoviricetes* of phylum *Uroviricota* of kingdom *Heunggongvirae* of realm *Duplodnaviria* of *Viruses* (https://ictv.global/taxonomy).

### Restriction endonuclease analysis

**Figure 5.**
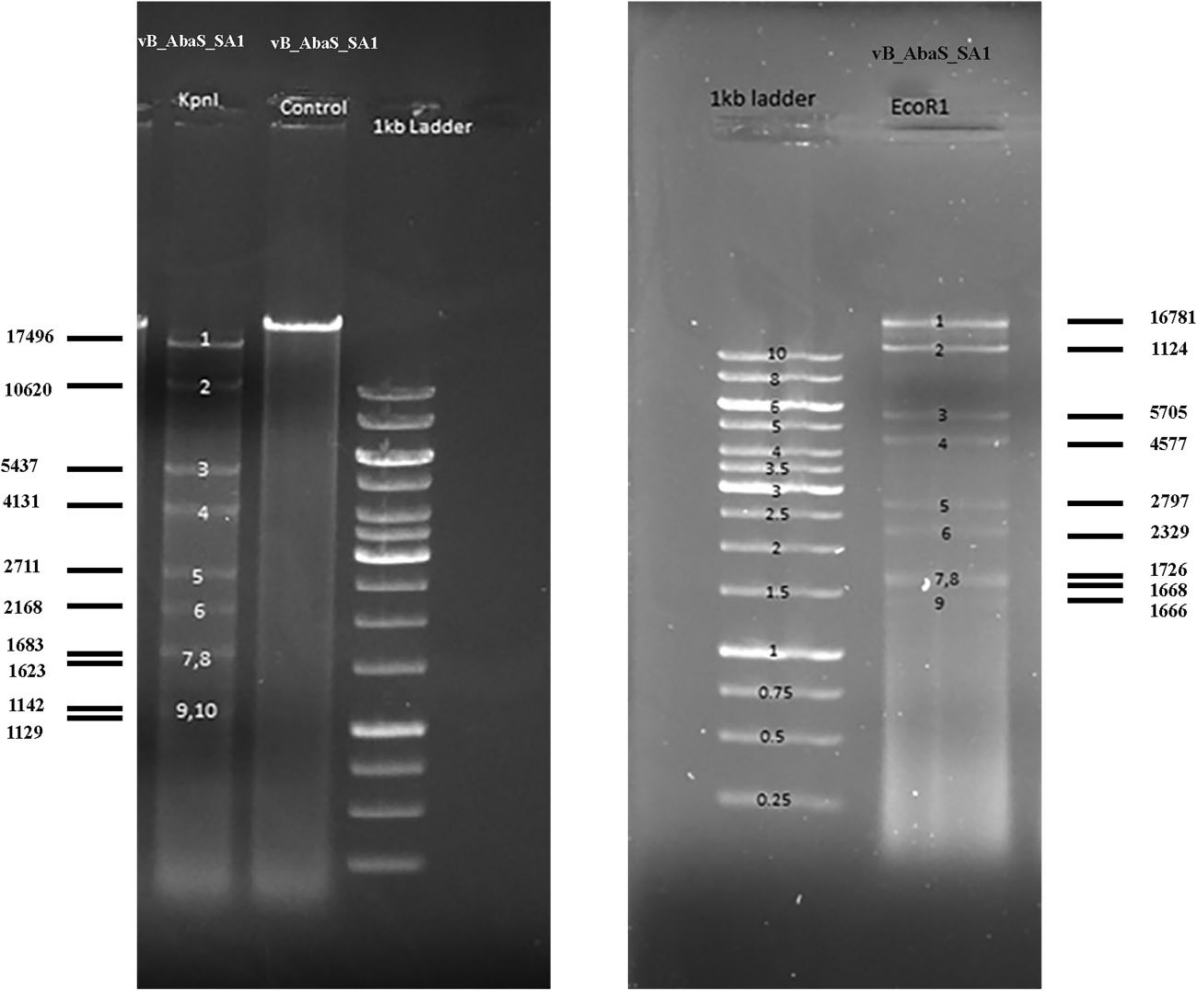
Restriction digestion results of vB_AbaS_SA1 with EcoR1 and KpnI. The *in silico* predicted restriction fragment sizes are indicated to the left and right of the gel images.

To verify the sequencing and assembly, the phage vB_AbaS_SA1 genomic DNA was digested with EcoRI and KpnI, and the pattern of the fragments was separated and observed on an agarose gel (Fig. 5). The results were in complete agreement with the genomic data, validating the correctness of the assembly.

### The combined effect of phage SA1 and antibiotics against *A. baumannii* isolates is synergistic

In the study, it was found that when phage SA1 was combined with meropenem, seven out of ten isolates showed a reduction in MIC by at least 4-fold. Similarly, when phage SA1 was combined with ampicillin-sulbactam, six isolates showed a 4-fold or higher reduction in MIC concentration. Additionally, the combination of phage SA1 with colistin showed a 4-fold or higher reduction in MIC in four isolates. At least a 2-fold reduction of MIC was observed in all the 10 phage-sensitive isolates after phage-antibiotics combination (Table 1).

**Figure 6.**
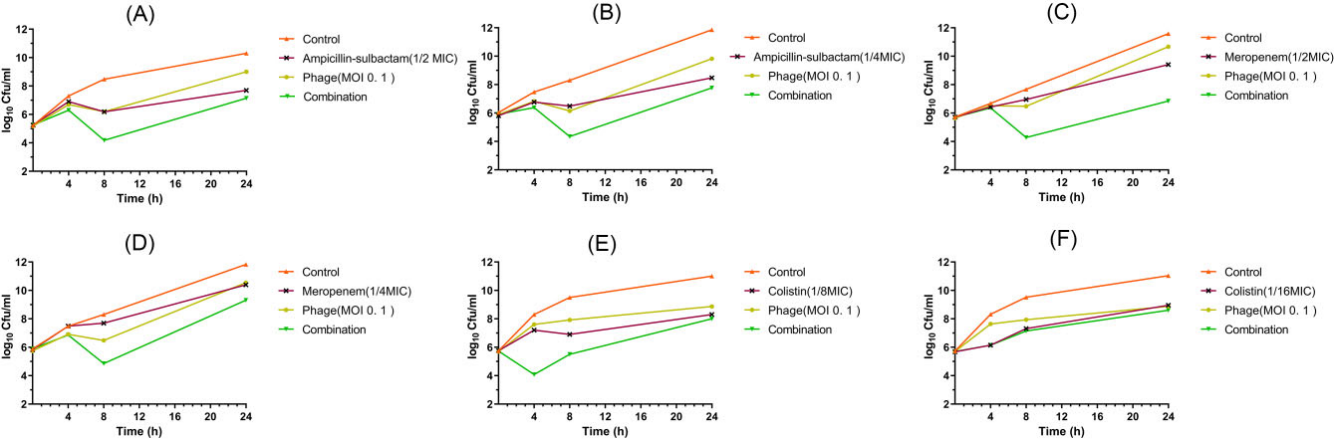
Time-kill experiment of phage alone and in combination with antibiotics (Ampicillin/sulbactam, Meropenem, and Colistin): (A) ½ × MIC Ampicillin/sulbactam (*A. baumannii* strain Ab1), (B) ¼ × MIC ampicillin/sulbactam (*A. baumannii* strain Ab1), (C) ½ × MIC meropenem (*A. baumannii* strain Ab6), (D) ¼ × MIC meropenem (*A. baumannii* strain Ab6), (E) 1/8 × MIC colistin (*A. baumannii* strain Ab2), (F) 1/16 × MIC colistin (*A. baumannii* strain Ab2): synergy was defined as a 2-log_10_ CFU/ml kill compared to the most effective agent (or double-combination regimen) alone at 24 h. Bactericidal activity was defined as a 3-log_10_ CFU/ml reduction from baseline. Data are presented as the mean ± standard deviation (SD).

The results of the time-kill method are demonstrated in Fig. 6. As seen, no synergy was observed in the wells containing meropenem at the concentrations of ½ and ¼ × MIC before 8 h; however, a synergistic effect was noted after 8 and 24 h of incubation when the meropenem concentration was at ^1^/_2_ × MIC. Despite this, no bactericidal activity was observed for this compound. In the case of the ampicillin-sulbactam and phage SA1 combination on isolate Ab1, a synergistic effect was observed at ½ × MIC concentration 8 h post-incubation. For isolate Ab2, a synergistic effect was evident 4 h post-incubation at ^1^/_8_ × MIC concentration of colistin. However, no effects were observed at ^1^/_16_ × MIC.

## Discussion

As a critical health priority, expanding research and developing new therapeutic strategies against *A. baumannii* are urgently needed (Butler et al. 2022). The control of *A. baumannii* is complicated due to its inherent and developed resistance to antibiotics and its ability to endure extended exposure to UV light, disinfectants, desiccation, and detergents (Jansen et al. 2018). This pathogen is increasingly recognized as a significant cause of global mortality, with mortality rates ranging from 30% to 76% reported in recent years (Wong et al. 2017). Our study aimed to determine the antimicrobial effectiveness of combining subinhibitory concentrations of ampicillin-sulbactam, meropenem, and colistin with an *A. baumannii-*specific bacteriophage.

Phage vB_AbaS_SA1, isolated from hospital effluent based on its capacity to form clear plaques, was able to infect ten of the 30 clinical MDR *A. baumannii* isolates. Electron microscopy revealed that phage SA1 has the siphovirus morphology characteristic of members of the class *Caudoviricetes* (bacterial and archaeal viruses with head-tail morphology). This observation was supported by its genome similarity to other phages of the genus *Vieuvirus* with 96.29% identity to *Acinetobacter* phage A2490.3, 95.27% to *Acinetobacter* phage A3926.3, 95.11% to *Acinetobacter* phage A1626.2, and 93.42 to *Acinetobacter* phage Acba_1 (Kornienko et al. 2020, Mardiana et al. 2022).

The SA1 burst size and latent period of 250 PFU/cell and 20–22 min, respectively, are very similar to those of another *A. baumannii* siphovirus, a latent period of 196 PFU/cell and 30 min (Mardiana et al. 2022). A myovirus specific to *A. baumannii*, on the other hand, had a latent period of 20 min and a burst size of 120 PFU/cell (Kusradze et al. 2016). Furthermore, phage D0, another siphovirus, had a latent period of 40 min and a burst size of 39 PFU/cell (Yuan et al. 2019). These investigations revealed that *Acinetobacter-*specific phages can belong to different families and exhibit diverse characteristics (Bagińska et al. 2023). Several key factors, such as the timing of inoculation, the rate of phage absorption to the host, and the burst size, significantly influence the effectiveness of phage therapy (Weld et al. 2004).

The host range of phages to infect clinical isolates of *A. baumannii* has varied in previous research, with some studies reporting broad host ranges and others more limited ones (Lin et al. 2010, Yang et al. 2010, Jin et al. 2012, Torabi et al. 2021, Kolsi et al. 2023). A broad host range is preferred for phages to be effective in phage therapy, as the phage can infect and lyse a diverse range of bacterial isolates. In general, a narrow host range has been typical for *A. baumannii* phages (Rastegar et al. 2024). Phage SA1 demonstrated a rather broad host range as it infected 33% of the MDR *A. baumannii* isolates. This is in contrast to a study that isolated and characterized three novel *A. baumannii* siphoviruses, fEg-Aba01, fLi-Aba02, and fLi-Aba03, that could only infect one of the 20 clinical strains tested (Badawy et al. 2020). Phages ZZ1 and AB1, on the other hand, infected 13% and 20% of the tested strains, respectively (Lin et al. 2010, Yang et al. 2010, Jin et al. 2012). The diversity of the tested strains should be known when determining the host range. In studies that tested host range on diverse characterized strains, the phage host range has usually been narrow, while in those experimenting on local uncharacterized strains, the reported host ranges have been wide. The host range of 33% for phage SA1 may prove more narrow as the diversity of the tested strains was not known and as the bacterial strains originated from the same location from where the phage was isolated (Yang et al. 2010, Kornienko et al. 2020, Oliveira et al. 2021, Huang et al. 2023, Kolsi et al. 2023).

The EOPs of SA1 were determined for 10 MDR *A. baumannii* isolates. Five isolates had high, one had moderate, and four had low EOP values. The outcomes suggested that phage SA1 may have the potential for application in phage therapy, as it is highly effective in destroying half of the sensitive strains. Compared to other studies, the results showed similarities and differences in EOP values. According to the results of Wintachai et al. when EOP was done on nine MDR *A. baumannii* isolates, three, four, and two isolates exhibited high, moderate, and low EOP values (Wintachai et al. 2022). Another investigation involved testing the EOP against 16 clinical isolates of *A. baumannii*, which exhibited high, moderate, and low EOP values in seven, six, and three strains, respectively (Wintachai and Voravuthikunchai 2022). The differences in EOP values in different studies might be attributed to the variability in phage and host strains (de Melo et al. 2019).

Findings in recent achievements in the treatment of infections caused by MDR *A. baumannii* by the topical and systematic administration of various phages in combination with antibiotics once again underscore the potential of bacteriophages as promising alternatives against bacterial infections that are difficult to treat otherwise (Zhou et al. 2018). In addition, using a combination of phages and antibiotics has been shown to enhance the potential of phage therapy in treating MDR bacterial infections. Several studies have shown an enhancement in the antibacterial effects of antibiotics and phages when combined compared to using each agent alone. Additionally, resistance developed less frequently with dual therapy because the second could eliminate a strain non-susceptible to one agent (Viertel et al. 2014).

PAS, the synergistic effect of phage and antibiotics in MDR bacterial infections has been the subject of several studies. PAS depends on the type of applied antibiotics, phages, and bacterial strains (Breidenstein et al. 2011). Our research revealed that a combination of phage and each of the mentioned antibiotics resulted in a minimum 2-fold decrease in the MICs of the antibiotics. Specifically, when the phage and meropenem were combined, the MIC of 70% of isolates decreased by at least 4-fold. This decrease was 60% for ampicillin/sulbactam and 40% for colistin. Therefore, the PAS should be determined for each particular combination. However, methods for PAS significantly vary, and it is not easy to compare results obtained by different authors (Gu Liu et al. 2020, Holger et al. 2022). In addition, PAS is rarely confirmed by the time-kill curve method, which is the gold standard in studying synergy. The time-kill curve is rarely used because the experimental procedure is robust and complex, requiring working with larger volumes and continuously counting bacteria at short intervals (Nikolic et al. 2022). We conducted a time-kill test to determine whether the combination of phage exhibits a synergistic relationship with the stated antibiotics. The results of the time-kill tests indicate that the combination of phage SA1 and certain antibiotics can have a synergistic effect on bacterial isolates. Our results suggest that the effectiveness of the combination of phage SA1 and antibiotics can vary depending on the type of antibiotic and its concentration. Further research is needed to fully understand the mechanisms behind these interactions and optimize the use of phage-antibiotic combinations in treating bacterial infections. Our findings on the synergistic effect of phage SA1 and meropenem at ½ × MIC align with the synergistic effect observed by Jansen et al. between the KARL-1 phage and meropenem. During their research, they discovered that KARL-1, a T4-like myovirus, exhibited significant synergy with meropenem but a more modest synergy with colistin on MDR strains of *A. baumannii* (Jansen et al. 2018). Additionally, the study conducted by Styles et al. found a synergy between the phage AbaM_PhT2 and colistin (Styles et al. 2020). Blasco et al. produced a mutant lytic phage (Ab105-2φΔCI) by deleting the CI repressor gene from the lysogenic phage Ab105-2phi. Their study showed *in vitro* synergistic antimicrobial effects against *A. baumannii* isolates, where a reduction of 4–7 log CFU/ml was observed when the combination of Ab105-2φΔCI mutant at 0.1, 1, and 10 MOI was used with meropenem at ^1^/_4_ and ^1^/_8_ × MIC (Blasco et al. 2019). According to Weber et al. combining ampicillin/sulbactam and phage at different concentrations resulted in complete bacterial elimination. These results indicate that using ampicillin/sulbactam and phage in combination could be a potentially effective treatment approach for MDR bacterial infections (Weber et al. 2020).

Schooley and colleagues successfully treated an MDR *A.baumannii* infection in a diabetic patient using personalized phage therapy. Nine different phages were administered, leading to the patient’s recovery and clearance of the infection (Schooley et al. 2017). The outcomes of this study have provided additional evidence to support the potential clinical efficacy of the strategy. However, the precise mechanism of immune modulation using the combination of phage SA1 and antibiotics *in vivo* remains uncertain and warrants further investigation. This study observed that the cell wall (for meropenem and ampicillin/sulbactam) and cell membrane (for colistin) are the targets of the bactericidal effect of the three examined antibiotics. However, the exact mechanism of the PAS response resulting from the combination of phage and these three antibiotics is yet to be fully understood. A better comprehension of the antibiotic selection and PAS mechanism is crucial in providing effective guidance for the clinical use of combined phage and antibiotic therapy to treat MDR bacterial infections. Additionally, this strategy can potentially enhance the phage’s ability to kill bacterial strains previously resistant to elimination by phage (Luo et al. 2022).

The genome characterization showed that SA1 has a linear dsDNA of 50 108 bp and belongs to the genus *Vieuvirus* of class *Caudoviricetes*. An integrase gene in the SA1 genome suggested that SA1 is likely a lysogenic phage, and, indeed, a DNA database search showed that homologous prophages are common in *A. baumannii* genomes. One of the most important points in phage therapy is using a lytic phage that lacks integration genes in its genome (Gill and Hyman 2010). The use of next-generation sequencing allows us to select phages that do not risk transferring unwanted genes, such as endotoxins, thus avoiding issues related to horizontal gene transfer. Despite these challenges, it is important to recognize the immense potential of temperate phages as therapeutic agents. When engineered in their lysogenic state, temperate phages can be used in phage therapy by converting them into lytic phages, which can extend their lifetime in the circulatory system of mammals and modify genes to enhance their killing effect (Blasco et al. 2019, Al-Anany et al. 2021). In conclusion, the emergence of antibiotic-resistant strains of *A. baumannii* has led to exploring alternative therapies, such as phage therapy. The combination of antibiotics with phage therapy is more effective in reducing the bacterial load of *A. baumannii in vitro* than either treatment alone. The use of combination therapy may have significant implications in the treatment of *A. baumannii* infections and warrants further investigation. As noted, SA1 is not a lytic phage, which limits its use as a phage therapy agent; however, given that it showed potent antibacterial activity alone and in combination with antibiotics, it can be turned into a suitable candidate for phage therapy after removing the integration-related parts of its genome.

## Supplementary Material

ftae028_Supplemental_File
