# Supplementary material for: Characterization of bacteriophage vB_AbaS_SA1 and its synergistic effects with antibiotics against clinical multidrug-resistant Acinetobacter baumannii isolates
Source: Pathog Dis. 2024 Oct 21;82:ftae028. doi: 10.1093/femspd/ftae028 (PMC11536755; doi:10.1093/femspd/ftae028)
Supplement: ftae028_Supplemental_File [file ftae028_supplemental_file.docx]

**Tables**

**Table S1.** Phage host range determined by spot assay on clinical isolates of *A. baumannii*

| Phage Name |  |  |  |  |  |  |  |  |  |  |  |
| --- | --- | --- | --- | --- | --- | --- | --- | --- | --- | --- | --- |
|  | Ab1 | Ab2 | Ab3 | Ab4 | Ab5 | Ab6 | Ab7 | Ab8 | Ab9 | Ab10 | Ab11-Ab30 |
| SA1 | + | + | + | + | + | + | + | + | + | + | - |

+ Susceptible, - not susceptible

**Table S2**. Efficiency of plating (EOP) on *XDR A. baumannii* isolates

| Bacteriophage name | vB_AbaS_SA1 |
| --- | --- |
| Lysed strains in spot test assays | N=10 |
| High production (EOP ≥0.5) | 5 (50%) |
| Medium production, (0.1≤ EOP≤ 0.5) | 1 (10%) |
| Low production, (0.001< EOP <0.1) | 4 (40%) |

**Figure legends**


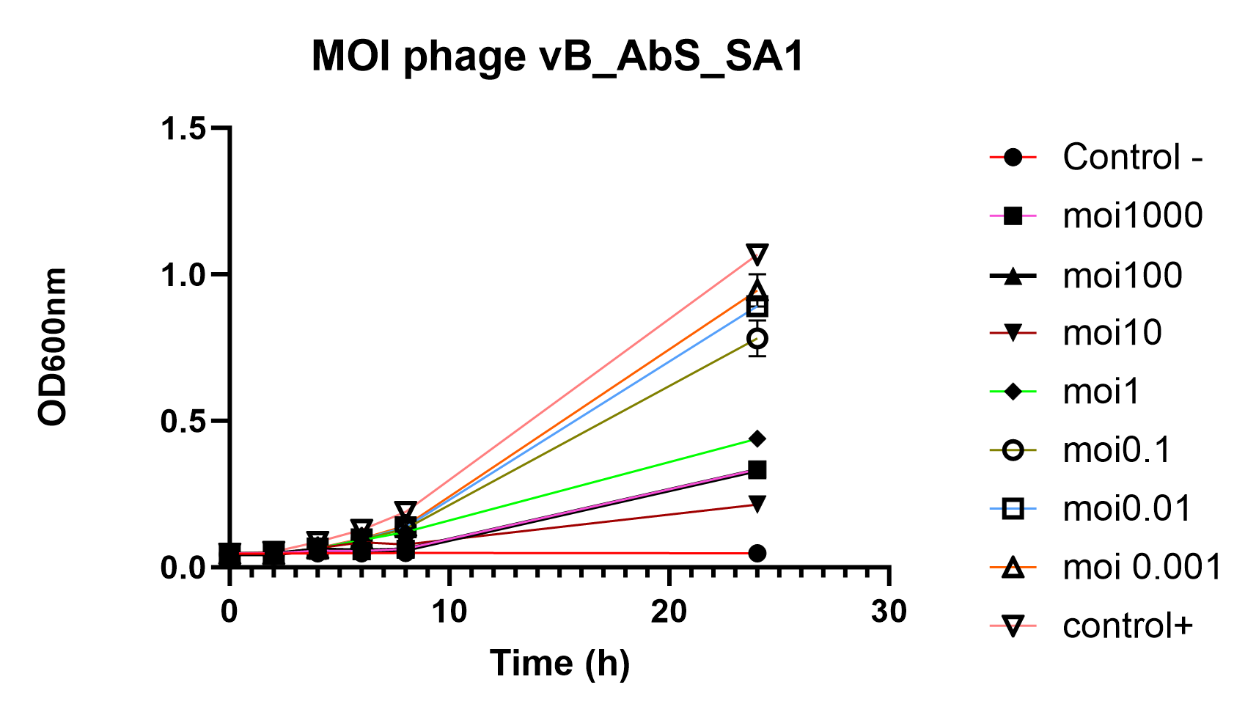


**Figure S1.** Determination of optimal MOI for vB_AbaS_SA1 against *A. baumannii* *in vitro*. *A. baumannii* isolate Ab8 was infected by vB_AbaS_SA1 at MOIs of 0.001, 0.01, 0.1, 1, 10, 100, and 1000 and cultured for up to 24 hours. *A. baumannii* Ab8, with the same volume of diluent was used as a positive control, and vB_AbaS_SA1 without bacteria in the same volume was used as a negative control. This experiment was repeated three times, and the data are shown as mean ± SEM.


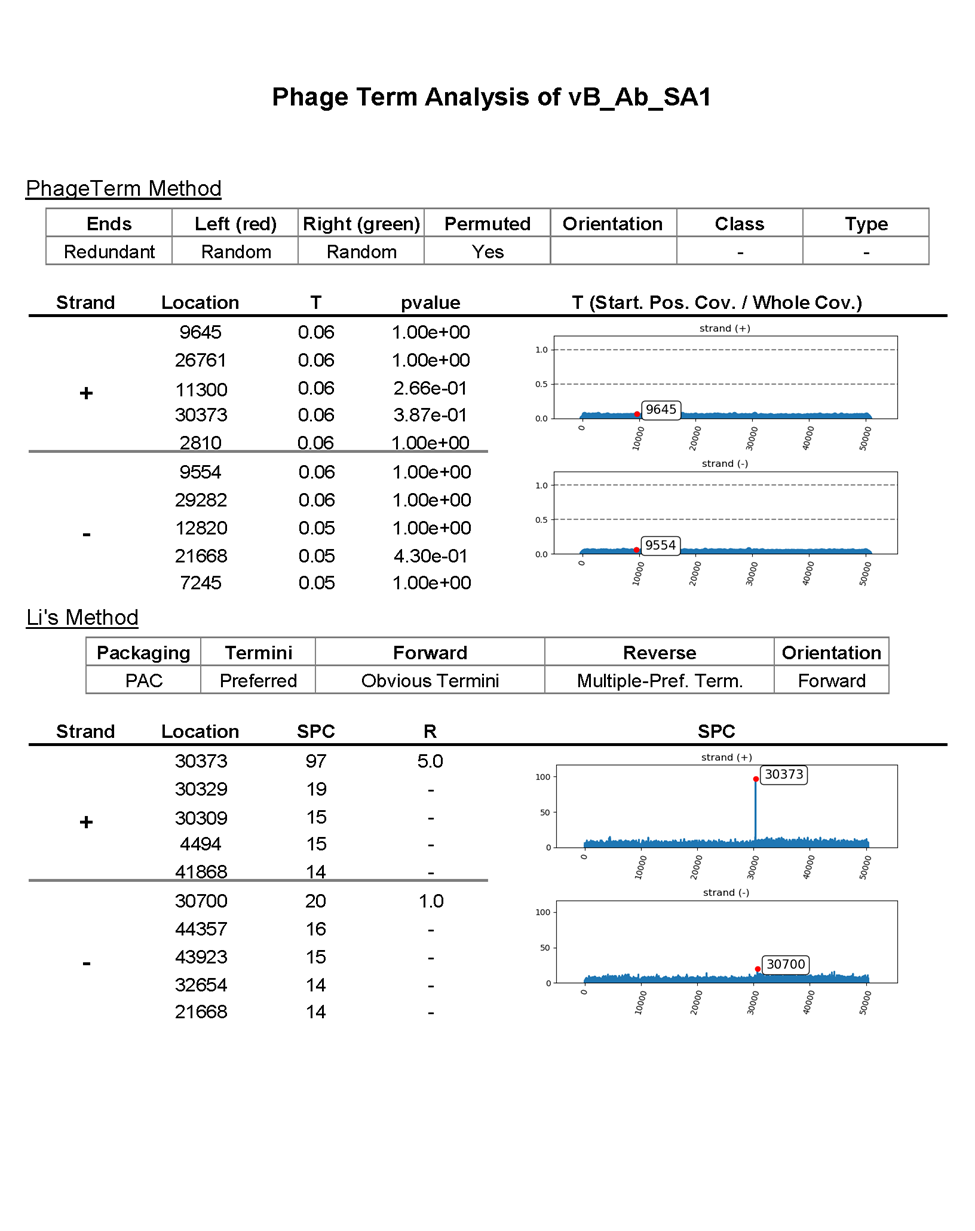


**Figure S2:** Determination of physical termini of vB_Abas_SA1 using Phage Term software
